# Metabolomic profiling of ovary in mice treated with FSH using ultra performance liquid chromatography/mass spectrometry

**DOI:** 10.1042/BSR20180965

**Published:** 2018-11-21

**Authors:** Liting Sun, Lu Chen, Yanwen Jiang, Yun Zhao, Fengge Wang, Xue Zheng, Chunjin Li, Xu Zhou

**Affiliations:** College of Animal Sciences, Jilin University, Changchun, P.R. China

**Keywords:** follicle, follicle-stimulating hormone, metabolomic profiling, ovary, UPLC/MS

## Abstract

The growth and development of follicles are a very complex physiological process that is regulated by endocrine, autocrine and paracrine mechanisms. The effect of small molecules in follicular microenvironment on follicular growth and development has not been clearly analyzed. In the present study, the metabolic changes in ovaries of FSH-stimulated mice were investigated. Metabolomic profiling of ovary stimulated by FSH were analyzed by ultra-performance liquid chromatography/mass spectrometry and characterized by principal components analysis and orthogonal partial least squares discriminant analysis. Multivariate statistical analysis identified 21 differentially metabolites in positive ion mode and 12 in negative ion mode in the FSH-treated mice compared with the control mice. These results indicated that various types of phosphatidylcholine were changed. Furthermore, the levels of L-Glutamyl 5-phosphate, N-Acetyl-L-aspartic acid, 4-fumarylacetoacetic acid, adenylylselenate and 5′-Methylthioadenosine in the ovaries of the FSH-stimulated mice were decreased. However, the levels of 19-hydroxytestosterone and 5,10-methenyltetrahydrofolic acid were significantly increased in the positive ion mode and negative ion mode, respectively. Thirty-three differential metabolites including fatty acid metabolism, amino acid metabolism and lipid metabolism in the ovaries of mice were affected by FSH injection. The findings of our study provide a new insight into understanding the follicular development.

## Background

The growth and development of ovarian follicles are a very complex physiological process that is regulated by endocrine, autocrine and paracrine mechanisms [1]. The importance of endocrine signals in the regulation of follicular development has long been known. It is generally believed that the follicle-stimulating hormone (FSH) acts primarily to promote proliferation of granulosa cells, follicular growth, expression of luteinizing hormone (LH) receptor and aromatization of androgens to estrogens [2]. Furthermore, the follicular microenvironment also plays a major role in determining the fate of follicles [3]. Molecules in follicular fluids have pivotal effects on follicular development. It is well known that insulin-like growth factor (IGF) promotes the synthesis of estradiol in antral follicles. In contrast, some low molecular-weight IGF-binding proteins have negative effects on the actions of IGFs by binding to them, such as IGFBP-2, IGFBP-4 and IGFBP-5. Meanwhile, it is well established that IGF and FSH work together to promote the production of estradiol [3]. In addition, inhibin A and B, vascular endothelial growth factor [4], lactoferrin [5], hyaluronan [6], leptin [7], 25-OH vitamin D, glucose and IL-8 [8,9], and many others molecules are also thought to be related to the growth and development of follicles.

Recently, metabolomics has been widely used for effectively addressing the roles of the small molecules (<10 kDa) in complex biological activities [10]. Metabolomics has gradually become a complementary technique to transcriptomics, genomics and proteomics. Based on liquid chromatography/mass spectrometry (LC/MS), gas chromatography/mass spectrometry (GC/MS) and nuclear magnetic resonance (NMR) spectroscopy, the major analytical platforms are used in metabonomics studies [11]. Among these techniques, as a peak resolution, high sensitivity and reproducibility analytical platform, ultra-performance liquid chromatography coupled to quadrupole time-of-flight mass spectrometry (UPLC-QTOF/MS) has been popularly relied on for the identification and quantification of metabolites [12]. The most metabolites and metabolic pathways among various species are similar. However, mRNAs, genes and proteins have diversity among different species. Therefore, metabolomics is a universal language describing the complex life activities of different species. Metabolites are possibly used to get more information than merely a direct detection of mRNAs (transcriptomes), gene expression (genomics) and proteins (proteomes). As a matter of fact, the gene activation with consequent mRNA and the synthesis of protein are not essentially associated with change of cellular function or morphology; nevertheless, metabolomes indicates the real functional status of the cell in biological system [13]. Small molecular-weight metabolites, as the final products of metabolism of cells, can illustrate all impacts affecting the development of follicle. There are numerous studies on the regulatory mechanism underlying the follicular development, but the changes of metabolic composition during follicular development have yet to be revealed. At the present stage, metabolomics is used to study the follicular fluid and the quality of oocytes for improving *in vitro* fertilization (IVF) [14]. And no metabolomic analysis has been performed on the developing ovary. Our metabolomic analysis of ovarian tissue in mice aims to explore a mechanism with regard to the development of follicles under FSH stimulation.

In the present study, the metabolic changes in the ovaries of FSH-stimulated mice were investigated. To our knowledge, this is the first study to analyze the follicular metabolomic changes in the ovaries of FSH-simulated mice based on UPLC-QTOF/MS. The present study revealed alterations in fatty acid metabolism, amino acid metabolism, lipid metabolism in the ovaries of mice simulated by FSH. It will be helpful to understand the mechanisms of the regulation of the growth and development of follicles.

## Materials and methods

### Animals and ovary collection

All animal experiments were approved by the Animal Protection and Utilization Committee of Jilin University. One hundred immature female (3 weeks old) BALB/c mice were achieved from the Medical Department of Jilin University, China. All mice were reared under conditions of controlled temperature (22–24°C) and humidity (60–70%), where they were given food and water *ad libitum* during the 12-h light/dark cycle. All mice were divided into two groups randomly as follows: mice in FSH group were injected intraperitoneally (IP) with FSH (10 IU/mouse; Ningbo Second Hormone Factory, Ningbo, China); mice in control group were injected IP with the same volume of vehicle (0.9% saline solution). After 48 h, the mice were all anesthetized with isoflurane and killed by cervical dislocation, and ovarian tissues were collected. Fifty milligrams of ovary tissue (20 ovaries) was collected for each sample and stored at −80°C until analysis. Five samples were measured for the control group and the FSH group, respectively.

### Specimen pretreatment

Approximately 50 mg ovary tissues were added to 800 μl of methanol. Then, all samples were grinded to fine homogenate using Grinding Millat 65 HZ for 90 s, vortexed for 30 s, centrifuged at 12000 ***g*** for 15 min at 4°C. Next, the supernatants (200 μl) were collected to a glass vial for LC-MS analysis.

### LC/MS analysis

All analyses were performed on a Ultra Performance LC system (Waters, U.S.A.) coupled to a Waters XevoTM G2 Q-TOF mass spectrometer (Waters MS Technologies, U.K.). The injection volume of each sample was 6 μl for each run. And, the mobile phase consisted of 0.1% (v/v) formic acid solution A and 0.1% formic acid in acetonitrile solution B. The gradient conditions were described in 5% acetonitrile for 0 to 1 min; 5–20% acetonitrile for 1 to 6 min; 20–50% acetonitrile for 6 to 9 min; 50–95% acetonitrile for 9 to 13 min; 95% acetonitrile for 13 to 15 min. The flow rate was 0.35 ml/min. Chromatographic separation was set at 40°C on an ACQUITY UPLC T3 column (2.1 mm × 100 mm, 1.8 μm).

Sample analysis was performed using positive or negative electrospray ionization (ESI) mode. For the ESI+ mode, the capillary voltage and the sampling cone were respectively set to 1.4 kV and 40 V. For the ESI− mode, the capillary voltage and the sampling cone were respectively set to 1.3 kV and 23 V. Mass spectrometry detections were operated in either the positive or negative ion mode with a cone gas flow of 50 l/h, desolvation gas flow of 600 l/h, source temperature of 120°C, desolvation temperature of 350°C, collision energy at 10–40 V, ion energy at 1 V scan time of 0.03 s, inter scan time of 0.02 s and the mass scanning range of 50–1500 m/z.

An equal volume (10 µl) of serum was mixed from each ovarian sample as a quality control standard (QCS). QC samples could demonstrate the reliability and stability of the UPLC-QTOF/MS system.

### Data processing and pattern recognition

Before the formal analysis, the data group was normalized to obtain more intuitive and reliable results. The goal of normalization was to make the scale of all variables (a certain digital feature, such as mean and standard deviation) at the same level. The raw data of LC/MS were transformed into a matrix including intensity, time and ion mass (m/z). Baseline correction and peak finding were performed by Progenesis QI data analysis software (Non-Linear Dynamics, Newcastle, U.K.). The resulting scaled datasets were applied to principal component analysis (PCA) firstly. Then, partial least-squares discriminant analysis (PLS-DA) and orthogonal partial least-squares discriminant analysis (OPLS-DA) were used by the SIMCA-P 11.0 version software (Umetrics AB, Umea, Sweden) to obtain clustering information and critical variables between the control group and the FSH group. When the predictive ability was indicated by Q^2^Y, the goodness of the fit was quantified by R^2^Y. MetaboAnalyst 3.0 platform (http://www.metaboanalyst.ca) was used to further analyze the resulting datasets. Metabolite peaks were identified by MS^E^ analysis and annotated with available biochemical online databases for instance METLIN (http://metlin.scripps.edu/), the Kyoto Encyclopedia of Genes, Genomes (KEGG, http://www.kegg.com/), and the human metabolite database (HMDB, http://www.hmdb.ca/).

In the present study, potential differential metabolites were selected based on the Variable Importance in the Projection threshold (VIP > 1). The *T*-test was carried out on measurement data, and *P*<0.05 was considered significant. All the data of metabolomics analysis were calculated by SPSS22.0 version software.

## Results

### Original chromatogram based on UPLC-QTOF/MS

**Figure 1:**
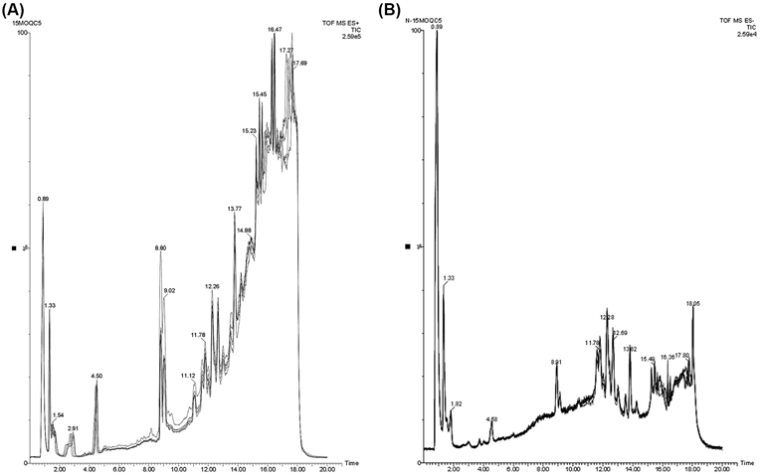
Representative total ion chromatogram of QC in positive ion mode (**A**, ESI+) and negative ion mode (**B**, ESI−)

**Figure 2:**
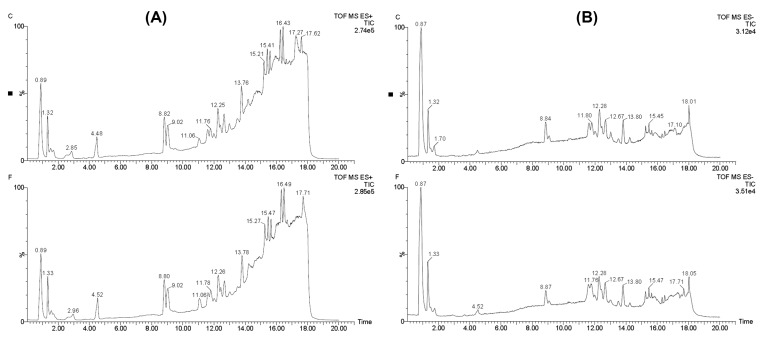
Total ion chromatogram of control group (C) and FSH (F) group. (**A**, ESI+; **B**, ESI−)

First, total ion chromatograms (TICs) of the QC samples in the positive or negative modes were analyzed by UPLC/Q-TOF MS, as shown in Figure 1. The good overlapping spectrum of all samples showed that the instrument has repeatability, retention time and stability. The typical TIC of the metabolic profiles of ovarian tissues from FSH-stimulated and control mice in the positive or negative modes were shown in Figure 2. Differences in peak intensities between the FSH group and the control group were observed.

### Multivariate statistical analysis

**Figure 3:**
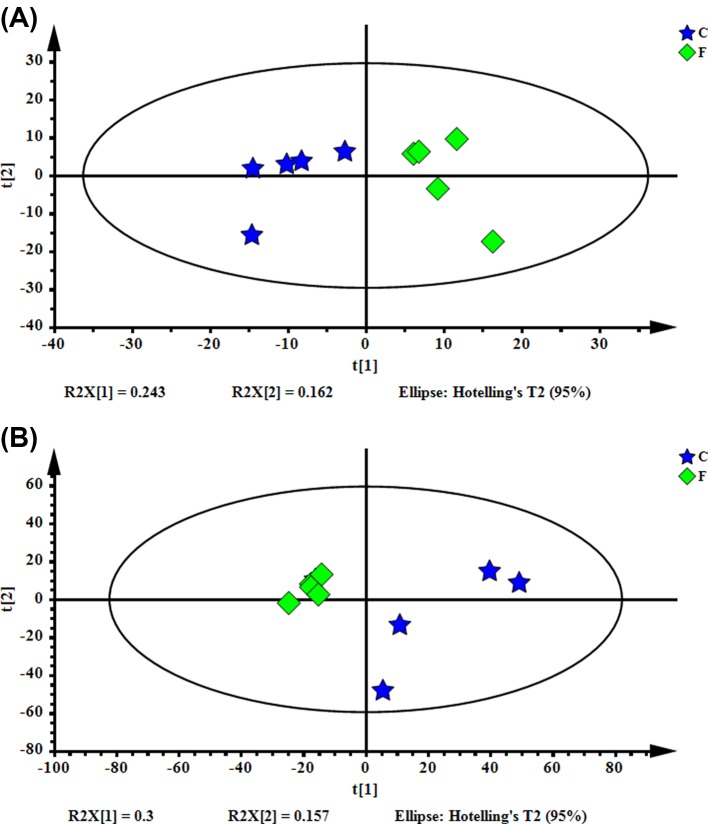
The PCA scores plot of the control group and the FSH group. (**A**, ESI+, R^2^X = 0. 457, Q^2^ = −0.0224; **B**, ESI−, R^2^X = 0.558, Q^2^ = −0.0591)

PCA models were used to fully understand the metabolic profile of the mouse ovary. The PCA scores showed that the samples from the FSH group and the control group indicated consistent classification. As shown in Figure 3A, there were two principal components in the positive ion mode and the PCA score plots were characterized by the following parameters: R^2^X = 0.457, *Q*^2^ = −0.0224. As shown in Figure 3B, there were three principal components in the negative ion mode and the PCA score plots were characterized by the following parameters: R^2^X = 0.558, *Q*^2^ = −0.0591. The results showed that the control group and the FSH group exhibited different metabolic characteristics. The outline view of all samples could be obtained by a PCA score plot. But, the specific changes in each group are still uncertain.

**Figure 4:**
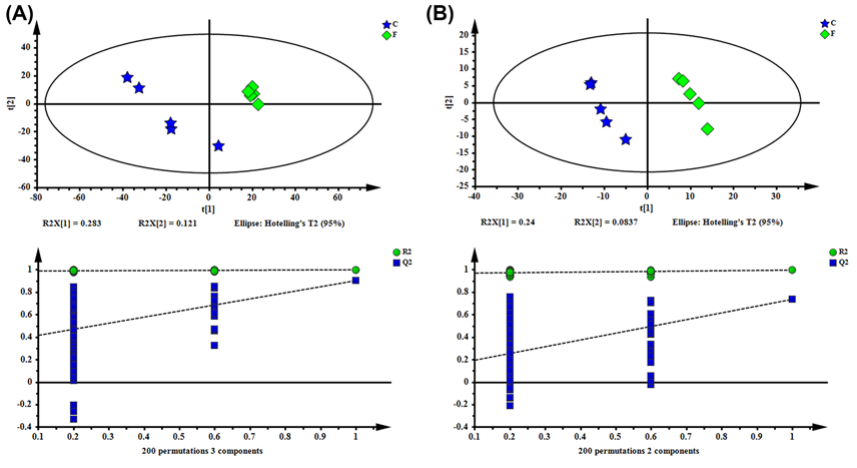
PLS-DA score plot of the control group and the FSH group (**A**, ESI+, R^2^X = 0.532, R^2^Y = 0.999, *Q*^2^ = 0.902; **B**, ESI−, R^2^X = 0.324, R^2^Y = 0.996, *Q*^2^ = 0.736). R^2^Y = The interpretation rate of the model, *Q*^2^ = The prediction rate of the model.

These differences were carried out the supervised multivariate analysis PLS-DA to track detailed differences between the two groups. In general, R^2^Y was an estimate of how good the model fits the Y data, and *Q*^2^ provided an estimate of how good the model predicts the X. In order to obtain a high predictive ability, the R^2^Y and *Q*^2^ values should be close to 1. The scores of PLS-DA plot (Figure 4) showed marked separation between the control and the FSH group. The R^2^X of the PLS-DA model in positive ion modes was 0.532, the R^2^Y was 0.999, and the *Q*^2^ was 0.902. The R^2^X of the PLS-DA model in negative ion modes was 0.324, the R^2^Y was 0.996, and the *Q*^2^ was 0.736. From the PLS-DA model parameters, the model was credible for interpreting the differences and the verification map did not show the phenomenon of ‘overfitting’ between the two groups. Therefore, these data could be used for subsequent analysis.

**Figure 5:**
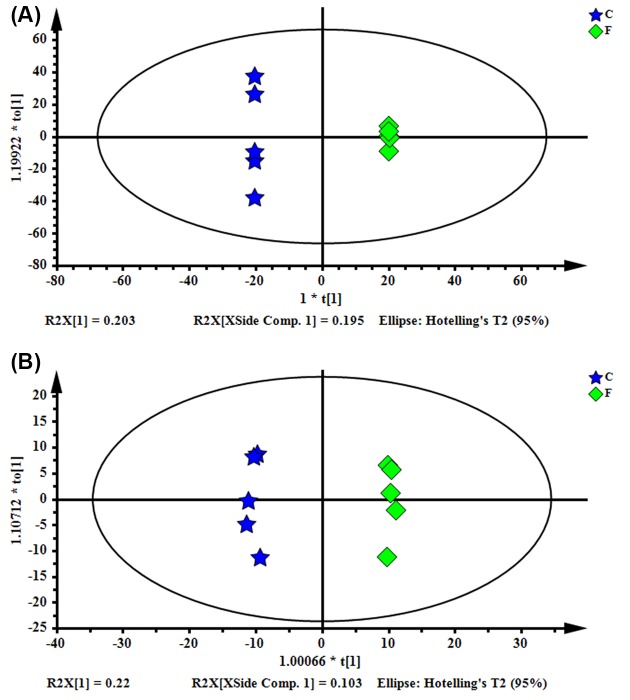
OPLS-DA score plot of the control group and the FSH group (**A**, ESI+; **B**, ESI−)

The OPLS-DA was used to better detect the metabolic variations in the ovaries of FSH-injected mice. As shown in Figure 5, the following parameters were R^2^X = 0.765, R^2^Y = 1, *Q*^2^ = 0.743 in positive ion modes and the following parameters were R^2^X = 0.324, R^2^Y = 0.996, *Q*^2^ = 0.794) in the negative ion modes. The OPLS-DA score plot demonstrated a clearer separation of the control group and the FSH group, and content modeling and predictability were achieved when the *Q*^2^ > 0.4.

### Identification of potential biomarkers and metabolic pathways

First, the variables with VIP value >1 have a good correlation with separation, which were applied to the candidate list. Then, to select significantly different variables (*P*<0.05), the *T*-test was performed in the following step. The comprehensive results showed that 33 differential metabolites could be annotated by searching the exact mass data (m/z) from the most intense peaks against the data of the online database: KEGG (http://www.kegg.jp/), METLIN (http://metlin.scripps.edu/) and HMDB (http://www.hmdb.ca/).

**Figure 6:**
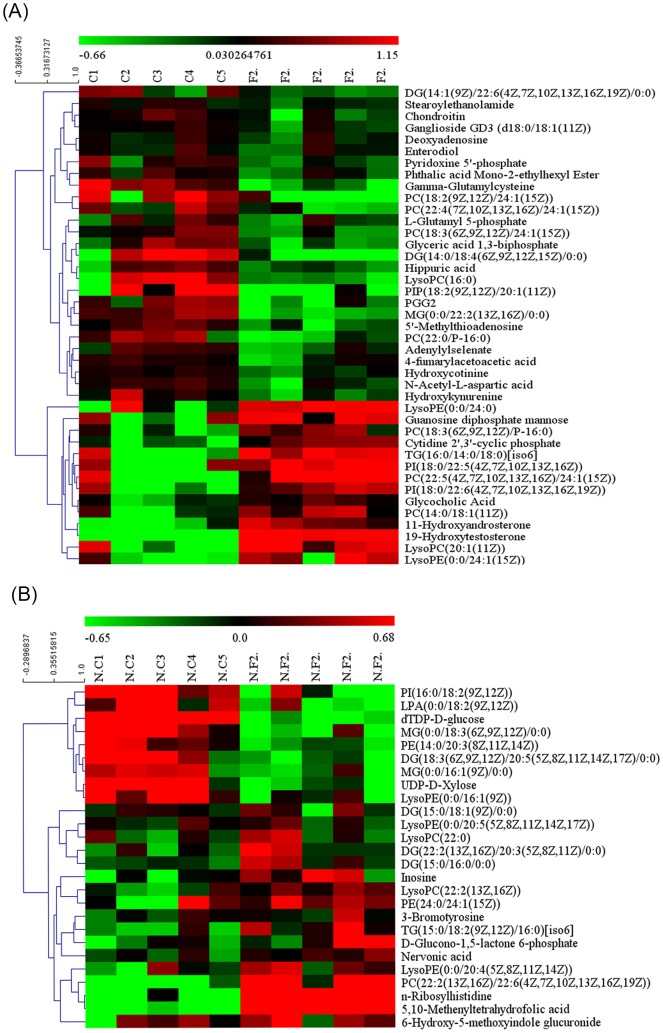
Heat map of differential metabolites Heatmaps representing the significantly changed metabolites between FSH group and the corresponding control group in ESI+ mode (**A**) and ESI− mode (**B**). In the figure, red indicates high content, green indicates low content, rows indicate differential substances, and columns indicate samples.

Based on analysis of LC/MS data, as shown in Tables 1 and 2 respectively, there were 21 marker metabolites in the positive ion mode and 12 metabolites in the negative ion mode. For unsupervised clustering, the significantly different metabolites were used to construct a heatmaps. The heatmaps were used to define differential metabolites in the FSH group and the control group that showed an obvious clustering for two ion modes in consistent with the OPLS-DA results in Figure 6. These results indicated that various types of phosphatidylcholine were changed. PC (14:0/18:1(11Z)), PC (18:3(6Z, 9Z, 12Z)/P-16:0), PC (22:5(4Z, 7Z, 10Z, 13Z, 16Z)/24:1(15Z)), PC (22:2(13Z, 16Z)/22:6(4Z, 7Z, 10Z, 13Z, 16Z, 19Z)), LysoPC (20:1(11Z)), LysoPC (22:0) and LysoPC (22:2(13Z, 16Z)) were increased, whereas the levels of PC (18:3(6Z, 9Z, 12Z)/24:1(15Z)), PC (18:2(9Z, 12Z)/24:1(15Z)), PC (22:0/P-16:0), PC (22:4(7Z, 10Z, 13Z, 16Z)/24:1(15Z)) and LysoPC (16:0) were decreased. To analyze the metabolic pathways that the changed phosphatidylcholines be involved in, linoleic acid metabolism, glycerophospholipid metabolism, arachidonic acid metabolism and α-inolenic acid metabolism were determined by MetaboAnalyst 3.0 platform. By the way, the results also indicated decreased levels of L-glutamyl 5-phosphate, deoxyadenosine, γ-glutamylcysteine, N-acetyl-L-aspartic acid, 4-fumarylacetoacetic acid, glyceric acid 1,3-biphosphate, adenylylselenate, prostaglandin G2 and 5′-methylthioadenosine in the ovary of the FSH-treated mice compared with the control in the positive ion mode. Besides, the levels of 19-hydroxytestosterone, glycocholic acid and guanosine diphosphate mannose were significantly increased in the positive ion mode. And, the results indicated decreased levels of PE (14:0/20:3(8Z, 11Z, 14Z)), UDP-D-Xylose and lysophosphatidic acid (LPA) (0:0/18:2(9Z, 12Z)) in the ovary of the FSH-treated mice compared with the control in the negative ion mode. The levels of inosine, nervonic acid, D-glucono-1,5-lactone 6-phosphate, 6-hydroxy-5-methoxyindole glucuronide, PE (24:0/24:1(15Z)) and 5,10-methenyltetrahydrofolic acid were significantly increased in the negative ion mode. These metabolites mainly contribute to the metabolic pathways of fatty acid, amino acid and lipid.

**Table 1:** The significantly different metabolites and pathway between control group and FSH group at ESI+

No.	RT (min)	Name	Molecular weight	VIP	*T*-test	Fold change (FSH/Control)	Pathway name
1	0.80	L-glutamyl 5-phosphate	227.0195	1.54	0.027	−0.10	Arginine and proline metabolism
2	9.29	Deoxyadenosine	251. 1018	1.60	0.019	−0.06	Purine metabolism
3	9.44	γ-Glutamylcysteine	250.0623	1.91	0.001	−0.32	γ-Glutamylcysteine
4	11.07	PC (14:0/18:1 (11Z))	731.5465	1.52	0.028	0.19	Glycerophospholipid metabolism; Linoleic acid metabolism; Arachidonic acid metabolism; α-Linolenic acid metabolism
5	11.66	Glycocholic acid	465. 3090	1.57	0.023	0.07	Primary bile acid biosynthesis
6	11.70	PC (18:3 (6Z, 9Z, 12Z)/P-16:0)	739.5516	1.47	0.036	0.16	Glycerophospholipid metabolism; Linoleic acid metabolism; Arachidonic acid metabolism; α-Linolenic acid metabolism
7	11.91	N-Acetyl-L-aspartic acid	175.0481	1.92	0.001	−0.14	Alanine, aspartate and glutamate metabolism
8	11.92	4-Fumarylacetoacetic acid	200.0321	1.71	0.009	−0.11	Tyrosine metabolism
9	12.12	Glyceric acid 1, 3-biphosphate	265.9593	1.57	0.022	−0.20	Glycolysis or gluconeogenesis
10	12.28	PC (18:3 (6Z, 9Z, 12Z)/24:1 (15Z))	865.6561	1.83	0.003	−0.17	Glycerophospholipid metabolism; Linoleic acid metabolism; Arachidonic acid metabolism; α-Linolenic acid metabolism
11	12.66	Adenylylselenate	474.9644	1.66	0.013	−0.13	Selenoamino acid metabolism
12	13.12	PC (22:5 (4Z, 7Z, 10Z, 13Z, 16Z)/24:1 (15Z))	917.6874	1.52	0.029	0.57	Glycerophospholipid metabolism; Linoleic acid metabolism; Arachidonic acid metabolism; α-Linolenic acid metabolism
13	13.17	Guanosine diphosphate mannose	605.0772	1.41	0.049	0.26	N-Glycan biosynthesis; Amino sugar and nucleotide sugar metabolism; Fructose and mannose metabolism
14	13.27	19-Hydroxytestosterone	304.2038	1.93	0.001	0.71	Steroid hormone biosynthesis
15	13.28	LysoPC (20:1 (11Z))	549.3794	1.55	0.025	0.33	Glycerophospholipid metabolism
16	13.38	Prostaglandin G2	368. 2199	1.68	0.012	−0.22	Arachidonic acid metabolism
17	13.80	PC (18:2 (9Z, 12Z)/24:1 (15Z))	867.6717	1.47	0.037	−0.36	Glycerophospholipid metabolism; Linoleic acid metabolism; Arachidonic acid metabolism; α-Linolenic acid metabolism
18	14.25	PC (22:0/P-16:0)	801.6611	1.73	0.008	−0.24	Glycerophospholipid metabolism; Linoleic acid metabolism; Arachidonic acid metabolism; α-Linolenic acid metabolism
19	16.05	PC (22:4 (7Z, 10Z, 13Z, 16Z)/24:1 (15Z))	919.7030	1.54	0.027	−0.17	Glycerophospholipid metabolism; Linoleic acid metabolism; Arachidonic acid metabolism; α-Linolenic acid metabolism
20	16.22	5′-Methylthioadenosine	297.0896	1.68	0.012	−0.17	Cysteine and methionine metabolism
21	18.11	LysoPC (16:0)	495.3325	1.44	0.042	−0.29	Glycerophospholipid metabolism

**Table 2:** The significantly different metabolites and pathway between control group and FSH group at ESI

No.	RT (min)	Name	Molecular weight	VIP	*T*-test	Fold change (FSH/Control)	Pathway Name
1	0.83	PE (14:0/20:3 (8Z, 11Z, 14Z))	713.4996	1.41	0.038	−0.11	Glycosylphosphatidylinositol (GPI)-anchor biosynthesis
2	1.35	Inosine	268.0808	1.48	0.026	0.20	Purine metabolism
3	1.52	Nervonic acid	366.3498	1.61	0.012	0.14	Biosynthesis of unsaturated fatty acids
4	7.57	D-Glucono-1,5-lactone 6-phosphate	258.0141	1.38	0.043	0.24	Pentose phosphate pathway
5	11.78	LysoPC (22:0)	579.4264	1.41	0.037	0.11	Glycerophospholipid metabolism
6	12.62	UDP-D-Xylose	536.0445	1.69	0.007	−0.33	Amino sugar and nucleotide sugar metabolism
7	13.05	LPA (0:0/18:2 (9Z, 12Z))	434.2433	1.44	0.032	−0.18	Glycerolipid metabolism
8	15.22	6-Hydroxy-5-methoxyindole glucuronide	339.0954	1.38	0.043	0.17	Pentose and glucuronate interconversions
9	15.78	LysoPC (22:2 (13Z, 16Z))	575.3951	1.70	0.006	0.15	Glycerophospholipid metabolism
10	15.81	PC (22:2 (13Z, 16Z)/22:6 (4Z, 7Z, 10Z, 13Z, 16Z, 19Z))	885.6248	1.89	0.001	0.44	Glycerophospholipid metabolism; Linoleic acid metabolism; α-Linolenic acid metabolism; Arachidonic acid metabolism
11	17.20	PE (24:0/24:1 (15Z))	913.7500	1.40	0.039	0.18	Glycosylphosphatidylinositol (GPI)-anchor biosynthesis
12	18.03	5,10-ethenyltetrahydrofolic acid	455.1553	2.12	0.000	0.79	One carbon pool by folate; Glyoxylate and dicarboxylate metabolism

## Discussion

Metabolomics, especially LC/MS-based metabolomics, is an emerging analytical tool in the research on components of follicular fluid, due to the comprehensive and quantitative measurement of many metabolic biomarkers in biological samples [14]. This metabolomics study based on UPLC/Q-TOF MS of ovaries of mice stimulated by FSH identified 21 marker metabolites in the positive ion mode and 12 metabolites in the negative ion mode. They represented fluctuations in multiple pathways, such as fatty acid metabolism, amino acid metabolism and lipid metabolism. Various types of phosphatidylcholine that are involved in glycerophospholipid metabolism were changed. LysOPC (16:0) is derived from the enzyme phospholipase A2 hydrolyzing phosphatidylcholine and is involved in the deacylation/reacylation cycle, regulating molecular species composition. In addition, phosphatidylcholine plays an important role in lipid signaling through the interaction of the lysophospholipid receptor (LPL-R) [15]. LPA is a phospholipid with a wide range of biological functions, such as cell proliferation, differentiation [16] and cell–cell interactions [17]. LPA is also a local factor regulating female reproductive function. Previous studies have documented that LPA plays a role in the reproductive systems of mice, sows, ewes and cows [18–21]. And a study reported the function of LPA as the local regulator of cow reproduction in follicular fluid [22]. Sinderewicz et al. [23] observed the probable link between LPA action and the factors related to the growth and development of bovine follicles, depending on the follicular type. While previous study has demonstrated that the lipids are bioactive compounds and relate to oocyte development [24]. In the present study, the LPA level was decreased by FSH stimulation. Thus, LPA may be involved in the development of ovarian follicles.

19-Hydroxytestosterone is an intermediate product of androgen and estrogen metabolism. It is produced from testosterone by the enzyme cytochrome P450 and then converted into 19-oxo testosterone [25]. In the present study, a higher level of 19-hydroxytestosterone was observed in the FSH group. FSH can activate aromatase in follicular granulosa cells. Thecal cells provide 19-hydroxytestosterone under the action of LH. Some substrates, like 19-hydroxytestosterone, enter the granulosa cells through the basement membrane and are converted into estradiol-17β by activated aromatase [26]. The theca interna of follicles produces only a small amount of estrogen. Estrogens synergized with FSH stimulation regulate granulosa cell proliferate, follicular fluid form and follicular cavity expansion, thereby modulate follicles growth and development [27].

Cyclooxygenase (COX), also known as prostaglandin synthase, has a very important role in the regulation of synthesis of prostanoids. Cyclooxygenase can catalyze the production of prostaglandin G_2_ (PGG_2_) from arachidonic acid. PGG_2_ can further generate prostaglandin H_2_ under the catalysis of peroxidase. Prostaglandin H_2_ can produce different end products under the action of downstream cell-specific enzymes, of which prostaglandin E_2_ (PGE_2_) is a kind of end product [28,29]. PGE2, as one of the key paracrine factors of LH pathway, is mainly expressed by the granulosa cells of the follicles at developmental stages [30]. PGE_2_ acts on the cumulus cells by binding with two kinds of receptors: the prostaglandin receptor 2 (PTGER2) and the prostaglandin receptor 4 (PTGER4) [31]. In the present study, PGG_2_ remarkably decreased in the FSH group compared with the control group. Lau et al. [32] showed that FSH/LH can promote PGE_2_ production in ovarian cancer cells at the protein and mRNA level through COX-1 and COX-2 up-regulation. Cai et al. [33] reported that PGE_2_ stimulates the expression of *Cyp19* in rat granulosa cells of preovulatory follicles. The study also suggests that the role of PGE_2_ is mediated by activation the cAMP/PKA pathway [34]. Activation of PTGER4 induces follicle development at a level comparable to that induced by FSH [34]. The present study supports these findings and suggests that PGG_2_ may contribute to the synthesis of PGE_2_ and may be indirectly involved in the growth and development of follicles in FSH-treated mice.

5,10-Methenyltetrahydrofolate (5,10-Methenyl-THF) is a form of tetrahydrofolate that is an intermediate metabolite of metabolism of one carbon pool by folate. In the present study, the level of 5,10-methenyltetrahydrofolic acid remarkably increased in the FSH-treated mice compared with that of control group. As one of important group B vitamins, folate is an essential nutrient for the body and is involved in various biochemical and metabolic reactions. Studies have shown that as an important methyl donor in the carbon metabolism cycle, folate has an important impact on pregnancy, pregnancy complications and birth defects. Further studies have shown that in the folate metabolic pathway, the gene polymorphism and folic acid metabolism of a key enzyme, 5,10-methylenetetetrahydrofolate reductase (MTHFR), play an important role in ovarian function. Gene polymorphism and the high homocysteine levels caused by MTHFR can lead to the damage of reproductive function and endocrine function, including follicular development, embryonic development and hormone secretion [35]. With the deep understanding of folate metabolism, MTHFR gene polymorphism may become a new genetic marker for predicting the risk of disease and a new target for related gene therapies [35]. The potential role of the MTHFR gene in regulating ovarian follicular activity is unclear. Rosen et al. [36] reported that MTHFR polymorphisms might associate with granulosa cells activity in growing follicles.

Bile acids are steroid acids found mainly in the bile of mammals and other vertebrates. Bile acid synthesis occurs in liver cells that synthesize primary bile acids by cytochrome P450-mediated oxidation of cholesterol. Bile acids also have hormonal actions throughout the body [37]. It is well known that cholesterol is high in human follicles, and it is used to form sex steroids [38–40]. Of particular interest, Smith et al. [41] provided the first evidence that the key enzymes of both the classical bile acid synthetic pathway and replaced synthetic pathways are present in oocytes and ovarian granulosa cells. Meanwhile, bile acids are produced by human follicular granulocytes in response to the presence of cholesterol in the culture medium in their study. In agreement with this finding, our study observed a decreased metabolite of glycocholic acid involved in primary bile acid biosynthesis pathway in FSH group. However, it is unclear whether bile acids are mainly synthesized by granulosa cells, oocytes or both. Further researches are needed on this aspect.

The present study indicates that the levels of deoxyadenosine and inosine involved in purine metabolism were changed. Purine metabolism plays an important role in energy metabolism [42]. There have been increasing interest in the purine metabolites and their effects in follicular fluid, since the 1980s. Lavy and colleagues studied the levels of purine metabolites in human follicles from both natural and stimulation cycles and noted that adenosine was an inhibitor of human oocyte maturation [43]. A recent study has reported the biochemical relationship between the levels of purines in follicular fluid and follicular/oocyte maturity in human [44]. In this study, the decreased deoxyadenosine and the increased inosine in the FSH group were found, suggested that purine metabolism was promoted by FSH stimulation.

## Conclusion

A metabolomic profiling of ovary in mice treated with FSH was studied based on UPLC Q-TOF/MS technique. As a result, 33 differential metabolites including fatty acid metabolism, amino acid metabolism, lipid metabolism in the ovaries of mice were affected by FSH injection. To our knowledge, the present study first investigates the effect of the FSH on metabonomic profiles in the ovary of mice, which is helpful to understand the mechanism in ovarian follicular development.

## Abbreviations

5,10-Methenyl-THF: 5,10-Methenyltetrahydrofolate
CE/MS: capillary electrophoresis/mass spectrometry
COX: cyclooxygenase
*Cyp19*: cytochrome P450, family 19
ESI: electrospray ionization
FSH: follicle stimulating hormone
GC/MS: gas chromatography/mass spectrometry
IGF: insulin-like growth factor
IVF: *in vitro* fertilization
LC/MC: liquid chromatography/mass spectrometry
LH: luteinizing hormone
LPA: lysophosphatidic acid
LPL-R: lysophospholipid receptor
MTHFR: 5,10-methylenetetetrahydrofolate reductase
NMR: nuclear magnetic resonance
OPLS-DA: orthogonal partial least squares discriminant analysis
PCA: principal components analysis
PGE2: prostaglandin E2
PLS-DA: principal components analysis
PTGER2: prostaglandin receptor 2
PTGER4: prostaglandin receptor 4
QCS: quality control standard
TIC: total ion current
UPLC-QTOF/MS: ultra performance liquid chromatography coupled with quadrupole time-of-flight mass spectrometry
VIP: variable importance in the projection
